# Stress and Burnout in Teachers During Times of Pandemic

**DOI:** 10.3389/fpsyg.2021.756007

**Published:** 2021-11-26

**Authors:** Natalia Vargas Rubilar, Laura Beatriz Oros

**Affiliations:** ^1^Instituto de Educación Superior N° 9-027, Universidad del Aconcagua, Mendoza, Argentina; ^2^Centro de Investigación en Psicología y Psicopedagogía-UCA-CONICET, Universidad Adventista del Plata, Mendoza, Argentina

**Keywords:** stress, burnout, teaching, pandemic, COVID-19

## Abstract

In Argentina, once mandatory isolation was declared due to the COVID-19 pandemic, teachers of all educational levels and modalities had to substantially modify their way of working. The aim of this study was to identify the work situations that education professionals perceived as threats under the modality of non-face-to-face teaching, and to describe the level of perceived stress and its possible effect on psychophysical symptoms. Likewise, it sought to examine possible differences in the manifestations of burnout depending on the level of perceived stress and associated symptoms. An empirical study with a cross-sectional design was developed, in which 9,058 Argentine teachers, who had to complete self-report measures, participated. The sampling method was non-random, using an online procedure of reclusion of volunteers. Descriptive techniques and non-parametric tests were used for data analysis. More than 60% of the educators reported high and moderately high levels of stress. The predominant stressors were uncertainty about the consequences of the pandemic, work overload and inadequate working environment. The more stress they perceived, the higher the manifestation of unwanted psychophysical symptoms. Professional burnout was higher for teachers with a higher load of stress and with more psychophysical indicators of discomfort. These results reveal the psychological impact of the COVID-19 pandemic on the education staff, and encourage the development of intervention measures to preserve the health of professionals.

## Introduction

The World Health Organization declared the outbreak of the new coronavirus a pandemic on March 11, 2020, after the number of people infected and deaths from COVID-19 increased exponentially in 110 countries on different continents. Given the aggravation of the epidemiological situation at an international level, this organization urged world leaders to take immediate measures in order to face this sanitary crisis. In Argentina, the National Executive Branch established the social, preventive and mandatory isolation, starting March 20, 2020, as a measure to prevent the contagion, mitigate the circulation of the virus and preserve public health. Consequently, face-to-face classes were suspended from that date at all levels and modalities. Facing this situation, educational institutions had to quickly reorganize in order to give continuity to the academic year under the non-face-to-face modality.

After the first weeks of mandatory quarantine, several journalistic reports highlighted that education professionals throughout the country manifested feelings of distress due to the closure of schools, and felt overwhelmed by the dizzying change that virtual teaching implied (Carnese, 2020; Di Vincenzo, 2020; Favant, 2020; Fernández, 2020; Figueroa Díaz, 2020; Santoro, 2020; Vallejos, 2020). In the same way, studies conducted by different teachers’ unions revealed that social confinement had substantially transformed the work scenario of educators. At the same time, they noticed that these changes were affecting the health of teachers of all levels (see Confederación de Trabajadores de la Educación de la República Argentina, 2020; Sindicato Argentino de Docentes Particulares, 2020; Sindicato Unificado de Trabajadores de la Educación de Buenos Aires, 2020).

Over the years, various authors have pointed out that education workers show a high risk of developing anxiety, stress and burnout as a consequence of being exposed to a wide range of work stressors in their daily activities (Esteve, 1994; Gil-Monte and Peiró, 1997; Schaufeli and Enzmann, 1998; Vandenberghe and Huberman, 1999; Manassero et al., 2003; Schaufeli, 2003; Gil-Monte, 2005; Menghi, 2015; Othman and Sivasubramaniam, 2019). In Argentina, since March of 2020, teachers have seen many aspects of their work modified. This situation could lead to an increase in the perception of work stressors and/or intensity educators attribute to those stressors, making them even more prone to those maladjustments.

The interactional model of stress, proposed by Lazarus and Folkman (1986), offers a conceptual framework that allows us to understand not only the background, but also the mediators and the possible consequences of psychological stress. According to this perspective, stress arises when the transactions with the environment are assessed as threatening. In this way, different situations and stimuli may assume the nature of stressors, as long as they are considered dangerous for the achievement or the maintenance of wellness. The greater the potential for damage perceived in the demands of the environment and the lower the ability of the individual to cope with them, the greater the negative impact of stress.

Demands are conceived as explicit or implicit pressures in the environment that lead to acting in a particular way. When these demands cannot be easily satisfied with available physical, psychological, social or material resources, and when demands come into conflict with personal goals, beliefs and expectations, they become a powerful source of stress (Lazarus, 2000).

Specifically in the occupational area, and following this line of thought, the World Health Organization defined burnout as the result of an imbalance between the demands and pressures of work, on the one hand, and the knowledge and abilities of workers, on the other hand (Leka et al., 2004). Regarding the educational context, various investigations have allowed the identification of those demands and working conditions that are habitually perceived as threatening and boosters of physical and psychological discomfort among teachers in non-isolation conditions. Among the most commonly signaled risk factors are: (a) behavioral problems, demotivation, absences and accidents suffered or provoked by students, (b) problems with the families of the students (criticism, complaints and lack of accompaniment to the student), (c) lack of support among colleagues, poor coordination and difficulties in teamwork, (d) administrative demands, conflict with superiors, injustices and low social and remunerative recognition, (e) work overload (multiple and excessive tasks to carry out in short periods of time and without sufficient breaks), (f) conflict and role ambiguity, (g) inadequate physical environment, lack of pedagogic material and lack of adequate equipment, (h) use of new technologies and (i) difficulties in combining work and family (Lambert et al., 2001; Salanova et al., 2003; Urquidi Treviño and Rodríguez Jiménez, 2010; Menghi, 2015; Goebel and Carlotto, 2019).

In the United States, a scale was created to assess the stress associated with different demands and resources peculiar to teaching work in primary schools based on the theoretical model of Lazarus and Folkman (1986) specifically (Lambert et al., 2001). The demands included behavior problems and student absences, administrative demands, a shortage of teaching materials, and so on. Among the resources, the availability and help of school support personnel, administrative support, community support and specialized training were assessed. The researchers noted a higher risk of occupational stress in those teachers who perceived high demands and low resources (Lambert et al., 2019).

The epidemiological situation framing the exercise of teaching today generates other concerns that are not inherent to the teaching role, but with a possible catalytic effect, such as fear of contagion, uncertainty about the duration of the pandemic and its possible impact on the economic situation, the physical distancing from social support networks, among others (MacIntyre et al., 2020; Sánchez Mendiola et al., 2020).

As a result, psychophysical manifestations of distress might emerge, with more or less severe consequences, depending on the case. Studies carried out with education professionals during the pandemic have reported an increase in headaches, muscle contractures, fatigue, anxiety, anguish, dizziness, lightheadedness, nervousness, and sleeping and eating disorders (Asociación del magisterio de Santa Fe, 2020; Confederación de Trabajadores de la Educación de la República Argentina, 2020; Sindicato Argentino de Docentes Particulares, 2020; Sindicato Unificado de Trabajadores de la Educación de Buenos Aires, 2020; Casali and Torres, 2021; Idris et al., 2021).

Moreover, it is known that when the teaching workload is perceived as higher than the resources available to meet it, the intention to continue practicing the profession, or choose it again if the opportunity arises, decreases significantly (Lambert et al., 2019). On the other hand, inadequate and long-term efforts to meet work demands can lead to burnout syndrome.

In current research on burnout syndrome, Maslach and Jackson’s (1981, 1986) definition is the most widely accepted by the scientific community and belongs to the multidimensional model they develop. According to these authors, burnout syndrome is an individual stress experience embedded in a context of complex social relationships that encompasses the concept the person has of themselves and others. Moreover, they defined it as a psychological syndrome of emotional exhaustion (i.e., drainage or reduction of emotional resources produced by interpersonal demands), depersonalization (i.e., development of negative, insensitive and cynical attitudes toward the recipient) and low personal fulfillment at work (i.e., tendency to negatively evaluate the work done) that can be developed in individuals in any type of activity whose object of work are people.

Gil-Monte and Peiró (1997) state that the burnout syndrome should be understood as a response to work stress that arises when the coping strategies the subject initially uses are not successful. The individual develops feelings of low personal fulfillment at work and emotional exhaustion by not being able to cope effectively with the stressors. Later on, the person displays behaviors of depersonalization as a new way of confrontation. Taking the transactional models as a reference, these authors recognize burnout as a variable that mediates between the stressors and their long-term effects. Within this context, burnout syndrome may be considered as an intermediate step in the stress-consequences of stress relationship.

Many studies have shown that stress and burnout are a potential problem within a wide range of occupations (Maslach et al., 2001; Schaufeli, 2003; Gil-Monte, 2005; Schaufeli et al., 2009). As regards teaching, studies conducted in different parts of the world have found that an important number of teachers are suffering from burnout syndrome (e.g., Fernández, 2002; Figueiredo-Ferraz et al., 2009; Rionda Arjona and Mares Cárdenas, 2011; Arias Gallegos and Jiménez Barrios, 2013; Ratto Dattoli et al., 2015), who show, in some cases, higher levels of burnout as compared to other occupations (e.g., Schaufeli and Enzmann, 1998; de Heus and Diekstra, 1999; Schaufeli, 2003; Johnson et al., 2005).

Although in recent years there has been interesting progress regarding the study of stress and burnout in Latin American teachers, new and more complex approaches are needed (Luy-Montejo et al., 2019). This research intends to expand the knowledge of these variables in a historical moment in which health care and well-being of educators are crucial. As it was expressed before, the conditions of isolation and social distancing imposed new potentially stressful and possibly more aversive challenges due to their cumulative effect that is associated to other factors typical of the pandemic. Given this context, it is urgent and necessary to know the levels of stress and burnout education professionals express. Mainly, because several studies portray the harmful consequences these maladjustments carry for the educators who suffer from them, for the students and for the organization where they work (see Maslach et al., 2001; Schaufeli, 2003; Schaufeli and Buunk, 2002). Considering this background, this study seeks to identify the working conditions that education professionals perceive as threatening in times of pandemic, and describe the level of perceived stress and its possible effect on psychophysical symptoms. Additionally, this study intends to identify differences in the manifestations of burnout according to the level of perceived stress and associated symptoms.

Based on the interactional model by Lazarus and Folkman (1986), significant differences are hypothesized in the experience of stress symptoms according to the level of perceived threat. The higher the perception of threat, the higher the cognitive deficit, the nervousness and the physical symptomatology. On the other hand, taking into consideration Maslach and Jackson’s (1986) classical approach (1986) and current research on stress and burnout syndrome in the teacher population (e.g., Esteras et al., 2016; Granados et al., 2019; Seijas-Solano, 2019), significant differences are hypothesized in the manifestations of burnout according to the perception of threat and the symptoms of stress. The higher the perception of threat and the symptoms of stress, the greater the burnout.

## Materials and Methods

### Type of Study and Design

An empirical, quantitative, *ex post*-facto, cross-sectional survey design was carried out (Calderón Saldaña and Alzamora de los Godos, 2018).

### Participants

In this study, 9,058 teachers residing in different Argentinian provinces participated. They worked in public or private education institutions, or both, in one of the four educational levels, in special or permanent education of youngsters and adults. The selection of participants was conducted through a non-randomized procedure of volunteer recruitment. Demographic and working characteristics of the sample are exposed in Table 1. The registered teachers completed all the required tests. The test basis was free of missing data.

**TABLE 1:** Demographic and working characteristics of the participants (*n* = 9.058).

**Variables**	**Frequency**	**Percentage**	**Mean**	***SD***
**Gender**				
Female	7,870	86.90		
Male	1,182	13.00		
Other	6	0.10		
**Age**			41.08	8.820
**Educational level or modality in which they perform**				
Kindergarden education	1,057	11.70		
Primary education	3,865	42.70		
Secondary education	2,592	28.60		
Higher education	349	3.90		
Special education	573	6.30		
Continuing education of young people and adults	622	6.90		
**Seniority in teaching**			12.85	8.390

*SD, Standard Deviation.*

### Instruments

In order to collect information about the demographic and working characteristics of the participants, closed questions were used, which revealed information regarding gender, age, educational level, work seniority and institutional management, among other aspects.

In order to analyze the situations that teachers perceive as stressful, a Scale of Teachers’ Stressors in Times of Pandemic (Oros et al., 2020) was used, which consists of 21 items, with five Likert-type answer options (Not stressful = 1, A little stressful = 2, Somewhat stressful = 3, Quite stressful = 4, Very stressful = 5). The items are factorially grouped into five factors: work environment and work overload (e.g., *Having little time to do all the tasks involved in remote work*), use of new technologies (e.g., *Learning how to use and master new technologies*), uncertainty about the duration and consequences of the pandemic for the teacher and the students (e.g., *Feeling uncertain toward the future, not knowing when the pandemic will end*), the organizational aspect of the educational institution (e.g., *Feeling that superiors do not understand the difficulties of working under these conditions*), and relationship with the students’ environment, conflict and role ambiguity (e.g., *Receiving multiple and simultaneous inquiries from students and/or parents*). The internal consistency values reported by the authors ranged between ω = 0.78 and 0.85 for the factors, being of ω = 0.93 for the full scale. In this study, the values obtained were: alpha = 0.85 for Work environment and work overload factor, alpha = 0.80 for Use of new technologies factor, alpha = 0.83 for Uncertainty about the duration and the consequences of the pandemic for the teacher and the students factor, alpha = 0.81 for Organizational aspect of the educational institution factor, and alpha = 0.77 for Relationship with the students’ environment, conflict and role ambiguity factor.

In order to know the symptoms of stress, the Scale of physical psychoemotional indicators of stress (Oros and Neifert, 2006) was administered. This scale consists of 22 items grouped in three dimensions: Cognitive deficit (e.g., “I find it difficult to focus,” “I forget things easily”) (alpha = 0.79), Nervousness (e.g., “I get nervous easily,” “I feel I worry excessively about everything” (alpha = 0.74) and physical symptoms (“I have insomnia or difficulty in falling asleep,” “I have neck and back pain” (alpha = 0.62). The items were answered in a Likert scale with five answer options (Never, Hardly ever, Sometimes, Often, Always). The internal consistency values for this study sample were: alpha = 0.84 for Cognitive deficit, alpha = 0.86 for Nervousness, and alpha = 0.74 for Physical symptoms.

Finally, in order to evaluate the burnout, a Spanish adaptation by Seisdedos (1997) of the Maslach Burnout Inventory (MBI), by Maslach and Jackson (1986) was used. This instrument consists of 22 items assessed with a Likert-type scale. Individuals indicate, through a range of seven adjectives, ranging from “Never” (0) to “Every day” (6), how often they experience each of the situations described. The factorization of the 22 items shows three orthogonal factors, which are called: (a) Emotional exhaustion, (b) Depersonalization, and (c) Personal fulfillment. The study of the MBI internal consistency in its original version in English showed, through the Cronbach alpha index, good values for each of the three subscales (0.90 for Emotional exhaustion, 0.79 for Depersonalization, and 0.71 for Personal fulfillment). In our country and region, this self-administered inventory has been used in various studies, showing pyschometric properties between moderately acceptable and satisfactory (e.g., Marucco et al., 2009; Malander, 2019). In this sample, Cronbach alpha values were very good for Emotional exhaustion (0.89) and Personal fulfillment (0.85), and fairly acceptable for Depersonalization (0.64).

### Data Collection Procedures

Due to the special conditions of social isolation in which this research was developed, the data collection was made through an online form. Volunteer recruitment was carried out through social media, electronic mail and digital messaging services, in some cases with the support of institutional and jurisdictional education authorities. The activity demanded approximately 15 min. The data collection started 46 days after the preventive social isolation was decreed in our country, in a period between April 27 and September 15, 2020. Teachers were provided with an email address for inquiries and an optional section at the end of the form to express opinions and comments.

### Statistical Procedures

The teachers’ answers were quantified and statistically processed. No missing data were recorded either at an item level or at a scale level.

To identify the situations valued as threatening, and the level of perceived stress, basic statistical analyses were conducted (calculus of means and standard deviations). In order to make descriptive comparisons between the different dimensions of the Stressor Scale, these means were considered by the number of items in each dimension.

The percentages of adherence to each answer section were calculated so as to estimate the general level of stress teachers perceived, taking into consideration the options of the Likert scale that were used in the evaluation (Not stressful = 1, A little stressful = 2, Somewhat stressful = 3, Quite stressful = 4, Very stressful = 5). In that way, scores weighted between 1 and 2 were considered indicative of Low level of stress, scores between 2.01 and 3 as indicative of moderately low level of stress, scores between 3.01 and 4 as moderately high, and scores over 4.01 as a high level of stress perception.

The assumptions of homoscedasticity and normality of the variables (univariate and multivariate) were also tested to determine the most adequate statistical test to study the hypotheses. For this purpose, the Kolmogorov-Smirnov, Box, Bartlett and Levene tests were used, from which the non-compliance with the mentioned assumptions was confirmed.

Therefore, with the purpose of analyzing possible differences among the psychophysical symptoms according to the level of stress, a non-parametric Kruskal Wallis *H* analysis was performed. Previously, the scores for each stressor were categorized in four groups (low stress, moderately low stress, moderately high stress and high stress) using the same criteria as with the general value. The Kruskal Wallis *H* test was also used to investigate the differences in the three manifestations of burnout based on perceived stress on the one hand, and psychophysical symptoms on the other. The psychophysical symptoms variable was categorized for this analysis using two cut points (< 33.33 = low symptomatology, 33.33–66.66 = moderate symptomatology, > 66.66 = high symptomatology).

The effect size for the *H* tests was estimated with the squared epsilon statistical (*E*^2^_*R*_), considering for its interpretation the Cohen’s criteria for the analogous test of partial eta^2^: 0.01 (small), 0.06 (moderate) and 0.14 (large). In all cases, *post hoc* contrasts were conducted through the non-parametric Mann-Whitney *U*-test.

### Ethical Considerations

The actions carried out in this work complied with the international ethical recommendations for research with human beings (i.e., Asamblea Médica Mundial, 2013; American Psychological Association, 2017). The teachers participated anonymously and voluntarily, stating their consent before answering the instructions on the form. No incentives of any kind were provided in exchange for participation. The information collected was treated confidentially, and was not accessed by persons outside the investigation.

## Results

### Situations Valued as Threatening and Level of Perceived Stress

According to the analysis of weighted means, of the five major stressors that were assessed, the preeminence of Uncertainty about the duration and the consequences of the pandemic is highlighted, followed in order of importance by Work environment and work overload, Relationship with the students’ environment, conflict and role ambiguity, Organizational aspect of the educational institution, and Use of new technologies factors (see Table 2).

**TABLE 2:** Descriptive information of the main variables.

**Variables**	***Min***	***Max***	***M***	***SD***	**Skewness**	**Kurtosis**
**Teachers’ stressors**						
Environment and work overload	1	5	3.37	1.09	–0.38	–0.83
Uncertainty	1	5	3.64	0.96	–0.54	–0.49
Organizational aspect	1	5	3.10	1.12	–0.03	–1.01
Relationships and role	1	5	3.14	1.12	–0.08	–0.95
New technologies	1	5	3.10	1.05	–0.05	–0.92
**Psychophysical symptoms**						
Cognitive deficit	0	28	11.03	5.27	0.14	–0.26
Nervousness	0	28	13.92	5.61	–0.12	–0.02
Physical symptoms	0	32	15.21	5.64	–0.07	–0.21
**Burnout**						
Emotional exhaustion	0	54	23.86	13.29	0.12	–0.91
Personal fulfillment	0	48	36.79	9.39	–1.05	0.76
Depersonalization	0	30	3.97	5.30	1.84	3.83

*Min, Minimum value of the subscale; Max, Maximum value of the subscale; M, Mean; SD, Standard deviation.*

On the other hand, statistical analyses revealed that 62.1% of teachers in the sample display general levels of stress between moderately high and high.

### Experience of Psychophysical Symptoms According to the Level of Stress

The analysis indicated significant differences in the psychophysical symptomatology according to the levels of perceived stress. The size of these differences was between moderate and large, the largest being the cognitive deficit. *Post hoc* contrasts showed significant differences among all the comparison groups, for all the variables included in the analysis (*p* < 0.001). The frequency with which physical symptoms, nervousness, and cognitive deficit are experienced increases significantly as the perception of stress increases (see Table 3).

**TABLE 3:** Results of the Kruskall Wallis test for the Psychophysical symptoms according to the Level of stress.

**Level of stress**	**Psychophysical symptoms**								
	**Cognitive deficit**			**Physical symptoms**			**Nervousness**		
	**AR**	**H**	**E^2^_R_**	**AR**	**H**	**E^2^_R_**	**AR**	**H**	**E^2^_R_**
**Environment and work overload**									
Low	2509.09	2020.33***	0.22	2855.21	1575.19***	0.17	2715.17	1860.83***	0.21
Moderately low	3627.56			3631.93			3533.57		
Moderately high	4780.37			4728.99			4766.98		
High	6007.08			5870.17			5976.23		
**Use of new technologies**									
Low	2999.28	1376.91***	0.15	3396.87	805.75***	0.09	3255.51	1028.28***	0.11
Moderately low	4129.24			4216.52			4137.52		
Moderately high	5068.12			4873.68			4978.98		
High	5978.46			5711.89			5823.75		
**Uncertainty about the duration and the consequences of the pandemic**									
Low	2766.42	1254.52***	0.14	3072.99	939.62***	0.10	2939.71	1077.14***	0.12
Moderately low	3403.51			3585.95			3508.31		
Moderately high	4457.98			4394.04			4413.40		
High	5593.49			5485.73			5539.48		
**Organizational aspect**									
Low	2999.83	1416.80***	0.16	3226.55	1056.38***	0.12	3140.00	1214.16***	0.13
Moderately low	4178.77			4210.08			4171.52		
Moderately high	5070.38			4978.76			5023.18		
High	5899.99			5737.15			5820.77		
**Relationships with the student environment, conflict and ambiguity of role**									
Low	3065.00	1287.06***	0.14	3262.33	1042.48***	0.12	3188.21	1170.02***	0.13
Moderately low	4173.73			4142.54			4118.58		
Moderately high	5049.78			4998.50			5026.35		
High	5860.07			5759.46			5832.59		

*AR, Average range; H, Kruskall Wallis test; E^2^_*R*_, Epsilon squared effect size.*

***p < 0.001.

### Manifestations of Burnout According to Level of Stress and Psychophysical Symptoms

The results revealed statistically significant differences in the three dimensions of Burnout depending on the level of stress. The size of the effect was larger for the Emotional exhaustion dimension (see Table 4). The *post hoc* contrasts showed significant differences among all the comparison groups for the dimensions of Emotional exhaustion and Depersonalization, whereas for Personal fulfillment a few pairs of non-significant average ranges were observed (see Table 5). In general, it is observed that as the level of stress increases, Exhaustion and Depersonalization increase, and Personal fulfillment decreases. An exception to this tendency was seen in Uncertainty about the consequences of the pandemic factor; in this case, both high and low levels of stress were associated with lower Personal fulfillment.

**TABLE 4:** Results of the Kruskall Wallis test of variance for Burnout according to the Level of stress.

**Level of stress**	**Burnout**								
	**Emotional exhaustion**			**Depersonalization**			**Personal fulfillment**		
	**AR**	**H**	**E^2^_R_**	**AR**	**H**	**E^2^_R_**	**AR**	**H**	**E^2^_R_**
**Environment and work overload**									
Low	2058.39	2913.39***	0.32	3929.62	215.34***	0.02	4818.84	32.44***	0.00
Moderately low	3435.64			4247.09			4624.82		
Moderately high	4906.87			4556.41			4465.53		
High	6254.87			5029.37			4371.97		
**Use of new technologies**									
Low	2903.12	1524.98***	0.17	4010.89	182.27***	0.02	4777.75	26.17***	0.00
Moderately low	4108.50			4342.48			4497.00		
Moderately high	5128.24			4776.39			4383.83		
High	6022.08			4999.18			4533.25		
**Uncertainty about the duration and the consequences of the pandemic**									
Low	2265.94	1644.81***	0.18	3891.83	210.14***	0.02	4338.18	22.19***	0.00
Moderately low	3400.45			4151.11			4744.48		
Moderately high	4436.88			4405.94			4569.48		
High	5732.11			4989.32			4426.19		
**Organizational aspect**									
Low	2580.40	2403.76***	0.27	3839.82	394.90***	0.04	4897.70	76.85***	0.01
Moderately low	4009.44			4211.83			4616.17		
Moderately high	5232.31			4859.60			4341.14		
High	6350.27			5238.77			4269.94		
**Relationships with the student environment, conflict and ambiguity of role**									
Low	2543.63	2371.27***	0.26	3902.01	340.87***	0.04	4782.79	43.20***	0.00
Moderately low	4015.91			4208.55			4622.50		
Moderately high	5286.08			4841.80			4414.93		
High	6305.07			5199.79			4291.45		

*AR, Average range; H, Kruskall Wallis test; E2_*R*_, Epsilon squared effect size.*

***p < 0.001.

**TABLE 5:** U de Mann Whitney values of the *post hoc* contrasts for Personal fulfillment according to the Levels of stress.

**Stress factors**	**L/ML**	**L/MH**	**L/H**	**ML/MH**	**ML/H**	**MH/H**
1	1379399*	1987614.5***	1823521***	2725458*	2500551**	3873727
2	2404464.5***	2379220.5***	1585064.5**	3538759.5	2322003.5	2298818
3	642825.5**	1178648*	1314508	2630342**	2760480.5***	5111892*
4	2431776***	2291782***	1790543***	2911322***	2275758***	2438424
5	2465516.5*	2443509.5***	1739315***	3111660.5**	2210118.5***	2405541

*L, Low; ML, Moderately low; MH, Moderately High; H, High; 1 = Work environment and work overload; 2, Use of new technologies; 3, Uncertainty about the duration and the consequences of the pandemic; 4, Organizational aspect of the educational institution; 5, Relationships with the student environment, conflict and ambiguity of role.*

*p < 0.05, **p < 0.01, ***p < 0.001.

Statistically significant differences were also observed in the three dimensions of Burnout according to the frequency of the psychophysical symptoms experienced. Again, the effect size was larger for Emotional exhaustion (see Table 6). The *post hoc* contrasts showed significant differences among all the comparison groups, for all the variables included in the analysis (*p* < 0.001). The higher the score in Cognitive deficit, Physical symptoms and Nervousness, the higher the score in Emotional Exhaustion and Depersonalization, but the lower the score in Personal fulfillment.

**TABLE 6:** Results of the Kruskall Wallis test of variance for Burnout according to the Level of symptoms.

**Symptoms of stress**	**Burnout**								
	**Emotional exhaustion**			**Depersonalization**			**Personal fulfillment**		
	**AR**	**H**	**E^2^_R_**	**AR**	**H**	**E^2^_R_**	**AR**	**H**	**E^2^_R_**
**Cognitive deficit**									
Low	2864.06	2975.03***	0.33	3859.85	535.62***	0.06	5248.29	503.27***	0.06
Moderate	4681.17			4557.81			4360.79		
High	6426.26			5321.55			3803.74		
**Physical symptoms**									
Low	3055.60	2055.72***	0.23	3980.93	259.37***	0.03	5337.08	528.64***	0.06
Moderate	4742.23			4722.83			4254.79		
High	6160.81			4991.77			3836.38		
**Nervousness**									
Low	2941.14	2468.81***	0.27	3878.74	383.32***	0.04	5365.19	606.29***	0.07
Moderate	4687.02			4721.97			4317.44		
High	6269.67			5106.17			3751.54		

*AR, Average range; H, Kruskall Wallis test; E2_*R*_, Epsilon squared effect size.*

***p < 0.001.

## Discussion

This study was conducted with the objective of: (a) identifying the workplace situations that teachers perceive as threatening in times of pandemic, (b) describing the level of perceived stress and its effect on the psychophysical symptoms, and (c) studying possible differences in burnout according to the level of perceived stress and associated symptoms. Hereinafter, the obtained results will be discussed, with reference to their adjustment degree with the previously stated hypotheses.

### Situations Valued as Threatening and Level of Perceived Stress

Of the five stressors assessed, uncertainty about the consequences of the pandemic for the teacher and the student stood out for its intensity (e.g., not knowing how the socioeconomic situation will continue, knowing there are students who do not have the technological resources to work remotely, being uncertain about the future, not knowing when the pandemic will end, and not knowing if students are understanding the contents). Preeminence of stressors associated with the working environment and work overload was observed (e.g., overlapping of work with household tasks, work schedule that is disorderly, unpredictable or different from the usual schedule, and lack of time to perform the tasks involved in remote work), as well as organizational aspects of the educational institution (e.g., receiving a greater number of requirements and demands from superiors), and the relationship with the students’ environment (e.g., receiving multiple and simultaneous inquiries from students and/or parents). As regards stress, it was noticed that 62.1% of educators presented high and moderately high levels of stress.

Similarly, studies conducted with Latin American teachers reported that they presented high levels of stress, anguish and anxiety during the suspension of face-to-face classes due to the global COVID-19 pandemic (Becerra Hernández, 2020; Casimiro Urcos et al., 2020). In Europe, under the same circumstances, Klapproth et al. (2020) found that German teachers experienced moderately high levels of stress on average. Likewise, they found that most educators pointed to the lack of technological equipment, internet connectivity, excessive workload and students’ demotivation as internal and external obstacles that made distance educational work difficult.

MacIntyre et al. (2020) examined work stressors that teachers who taught foreign languages in different educational levels and countries were exposed during school closings. These authors found that teachers identified work overload and concern for their family’s health as the most stressful. This was followed by loss of control over work, overlapping of work with household tasks, loss of control over personal decisions, irregular schedules and concern about finances. Coinciding with this study, they also highlighted that some educators mentioned not knowing when the pandemic would end as a source of stress.

In Argentina, between the months of April and June of 2020, different syndicates conducted studies in order to learn about aspects associated with the health and working conditions of educators of all educational levels and modalities, in the context of isolation and mandatory social distancing. These organizations reported that most teachers pointed out their work-related activities had increased and demanded more time. Consequently, they had to dedicate more hours to their daily work. A high percentage of educators reported not having adequate technological resources nor a comfortable isolated place to work at home. Moreover, combining work-related activities, household task and caring for their children, elderly or other family members was largely problematic for teachers. They also reported receiving communications outside working hours and contradictory instructions from educational authorities. Likewise, they revealed difficulties in supporting students’ schooling, mainly because many students did not have the necessary equipment and connectivity to work from home. Lastly, a significant number of educators expressed feeling concerned, anguished and suffering from stress (see Asociación del magisterio de Santa Fe, 2020; Confederación de Trabajadores de la Educación de la República Argentina, 2020; Sindicato Argentino de Docentes Particulares, 2020; Sindicato Unificado de Trabajadores de la Educación de Buenos Aires, 2020).

### Experience of Psychophysical Symptoms According to the Level of Stress

Results indicated that as the perception of stress increases, so do cognitive deficit, nervousness and various physical symptoms. These findings confirm the initial hypothesis and are consistent with the preliminary report by Arias et al. (2020). The authors observed that after 60 days of the onset of the quarantine in Argentina, teachers reported a significant increase in their workload and that this was associated to physical symptoms, especially lower back pain and discomfort in their arms and wrists. Similarly, Nieva’s (2020) study, conducted in Argentina during the context of social isolation due to COVID-19, registered a positive and significant correlation among various occupational stressors, somatization and other psychological symptoms in teachers. Likewise, 62% of Argentine teachers consulted by Casali and Torres (2021) expressed having worsened some psychophysical signs linked to stress in the context of the pandemic.

Similar results have also been documented before the onset of this sanitary contingency. For instance, Oramas Viera et al. (2007) assessed a sample of Venezuelan teachers, observing that the perception of stressors was positively and statistically significantly related to different functional symptoms (cognitive, affective, conative and psychosomatic). Comparably, Harmsen et al. (2019) found that the perception of stressors in teachers in the Netherlands kept a positive correlation with various stress responses, such as loss of pleasure, poorer sleep quality, fatigue, etc.

The general stress model proposed by Lazarus and Folkman (1986) and later taken up by Peiró and Salvador (1993) to explain work stress provides a solid frame for the interpretation of these results. According to their postulates, the stimuli that are perceived as threatening, in this case coming from the occupational context of teaching, produce a series of changes in the individual at a social, emotional and physiological level. These changes, with immediate and long-term effects, may produce suffering and deterioration of health and well-being (Ramos et al., 1997). Lazarus (2000) states that stress processes affect health in two ways. In the first place, they alter the neurochemistry of the body, that is, chemical and hormonal defenses that produce changes in sympathetic activity, secretion of catecholamines, etc., are activated as a reaction to stress (Krantz et al., 1985). Secondly, stress may favor the adoption of dysfunctional behaviors and coping styles. People exposed to situations perceived as stressful may attempt to obtain relief through the implementation of inadequate strategies, such as the consumption of alcohol, tobacco and other harmful substances, and excess or lack of food, which would derive in several psychophysical ailments.

On the other hand, some specific symptoms, such as those associated with the lower back, may be clearly explained by the large number of hours that teachers had to work sitting down in their own homes, possibly in an incorrect posture and/or using ergonomically inadequate furniture. Similarly, the excess of activities, the lack of time and the impossibility of leaving their residencies (due to the isolation dispositions) may have significantly increased sedentary lifestyles, also generating the appearance or exacerbation of these symptoms and others associated to the quality of sleep, to eating habits or to mental state (Bane et al., 2021).

Beyond the particular impact the stated situations may have had, it was probably the combination of all that explained the manifestation of various psychophysical symptoms presented when facing the stress involved in teaching in such particular contextual conditions.

### Manifestations of Burnout According to the Level of Stress and Psychophysical Symptoms

The results showed that as stress increases, so do Emotional exhaustion and Depersonalization, with Emotional exhaustion having a greater size effect. Regarding personal fulfillment, it was observed that, in general, it decreases as the perception of stress increases. It was also noted that the more teachers experience Cognitive deficit, Physical symptoms and Nervousness, the lower their Personal fulfillment, and the higher their Emotional exhaustion and Depersonalization. These results confirm the second hypothesis presented in this study.

Studies conducted with teachers before the COVID-19 pandemic was declared have reported results similar to those obtained in this study. For instance, a study with Venezuelan teachers found that Burnout syndrome was significantly correlated with the perception of stress and its symptoms. In this study, Emotional exhaustion also showed a higher correlation with perception of stress and psychophysical symptoms (Oramas Viera et al., 2007). In a similar way, a research conducted with Portuguese teachers found that the perceived stress was inversely proportional to Personal fulfillment and directly proportional to Emotional exhaustion and Depersonalization (Teles et al., 2020).

As stated before, burnout syndrome is a prolonged response to chronic stressors at work. It appears when the coping strategies used to handle work stressors are not functional. The individual, unable to mitigate or eliminate the source of stress, generates a series of physiological, cognitive, affective, attitudinal and behavioral responses that work as mediating variables between perceived stress and its consequences (Gil-Monte, 2005; Maslach, 2009). In this context, it is possible to think that teachers who experienced intense levels of work stress under the non-face-to-face teaching modality may have used ineffective coping strategies. In response, they possibly saw their emotional resources depleted, developed negative attitudes toward the students and experienced feelings of low competence and success at work.

It was surprising that, in reference to Uncertainty about the consequences of the pandemic for the teacher and the student dimension, both high and low levels of stress were associated with lower Personal fulfillment. This finding is partially dissenting with the starting hypothesis, and not easy to interpret, especially considering the lack of empirical background on the topic. It would be appropriate to deepen on this topic in future research.

## Conclusion, Recommendations and Limitations of the Study

In conclusion, it is possible to assert that a large proportion of Argentine teachers were significantly affected by occupational stress during the suspension of face-to-face classes. During this period, the stressors with a higher impact were associated with pandemic concerns and work overload, which, along with other situations perceived as threatening, explained the appearance of various psychophysical symptoms and burnout.

Based on these results, it would be desirable that national, jurisdictional and institutional education authorities critically reviewed the administrative protocols that were used during the so called first wave of cases, in order to adjust communication, planning, assessment and supervision procedures that have exceeded the actual capacities of many teachers (in terms of time, knowledge, technological skills, etc.). This review would be crucial to provide good quality education to students and offer better working conditions to educators, for as long as the pandemic lasts, schooling in Argentine educational institutions will be face-to-face, non-face-to-face or combined, depending on the epidemiological conditions.

At the same time, and beyond the measures educational institutions may carry out for the benefit of teachers, it is compelling that from health psychology instances that promote the psychosocial resources of teachers are generated. The results of this study could be capitalized to implement intervention measures (workshops, trainings, focus groups) aiming at reducing teacher discomfort and strengthening those resources that enhance the recovery and protection of their health and wellbeing. In this sense, it is especially recommended to strengthen teachers’ engagement, social support networks, existential beliefs and locus of control, social skills and functional coping strategies (Lazarus and Folkman, 1986). Similarly, it is suggested to offer seminars and guidance on how to optimize physical health and prevent discomfort, putting emphasis on, for instance, indications on correct body posture, good sleeping and eating habits, physical exercise, among others.

Although several months have passed since the beginning of the sanitary contingency, and teaching is no longer exclusively remote in formal education, it is possible that educators continue to perceive several demands as stressful, especially because of their changing nature. If no psycho-educational and therapeutic alternatives that allow the drainage of tensions are offered to educators, their psychophysical state may continue to deteriorate with serious long-term consequences, both at personal level (e.g., health loss, demotivation), and at organizational level (e.g., absenteeism, leaves, dropouts, decrease in the education quality).

This research presents some limitations that could be supplemented in future studies on this topic. Firstly, although the sample size was large, the lack of randomization restricts the generalization of the results. It would be desirable in future studies to extract random samples with representativeness from different regions of the country.

On the other hand, it should be noted that there are still no Argentine standardization studies that establish cut-off points for the interpretation of the variables analyzed here. For this reason, an *ad hoc* procedure based on sample frequencies and percentiles was used to categorize stress levels and psychophysical symptoms. In future studies, it is recommended to work on the development of normative values for the Argentine population.

In relation to the variables involved, this research has not included the analysis of coping, a variable that is crucial to deepen the understanding of stress and its possible consequences. It is recommended that its evaluation be included in future studies so as to determine how coping may mediate the relationship between perceived stress and its consequent burnout.

The cross-sectional nature of this study could also be considered a limitation, since no information is provided about the evolution of the perception of stressors, symptoms and burnout over time. This will be pending for future works.

## Data Availability Statement

The raw data supporting the conclusions of this article will be made available by the authors, without undue reservation.

## Ethics Statement

Ethical review and approval was not required for the study on human participants in accordance with the local legislation and institutional requirements. The patients/participants provided their written informed consent to participate in this study.

## Author Contributions

NV was the originator of the idea, organized the database, and wrote sections of the manuscript. LO contributed to the design of the study, performed the statistical analysis, and wrote sections of the manuscript. Both authors contributed to the data collection, read, and approved the final manuscript.

## Conflict of Interest

The authors declare that the research was conducted in the absence of any commercial or financial relationships that could be construed as a potential conflict of interest.

## Publisher’s Note

All claims expressed in this article are solely those of the authors and do not necessarily represent those of their affiliated organizations, or those of the publisher, the editors and the reviewers. Any product that may be evaluated in this article, or claim that may be made by its manufacturer, is not guaranteed or endorsed by the publisher.
